# Synthesis of mesoporous SiO_2_–CeO_2_ hybrid nanostructures with high catalytic activity for transamidation reaction†

**DOI:** 10.1039/d3ra01552a

**Published:** 2023-04-28

**Authors:** Manu Sharma, Harikrishnan K, Umesh Kumar Gaur, Ashok K. Ganguli

**Affiliations:** a Central University of Gujarat Gandhinagar India manu.sharma@cug.ac.in; b VP & RPTP Science College Vallabh Nagar India; c Indian Institute of Technology Delhi New Delhi India

## Abstract

Transamidation reactions catalyzed by boronic acid derivatives and metal catalysts are well known nevertheless their requirement for elevated temperatures and long reaction times were considered major obstacles in converting amides to *N*-alkyl amides with the coupling of primary amides and amines. The acidic–basic co-existence of ceria nanoparticles is considered a perfect choice for different catalytic activities. Mesoporous silica on the other hand is well known for its use as a supporting material for catalysts owing to its excellent characteristics like large surface area, good absorption capacity, and high-temperature stability. The SiO_2_–CeO_2_ hybrid nanocomposite was prepared by solvothermal route followed by annealing and the formation of the catalyst was confirmed by XRD, EDX, FTIR, and TEM characterization techniques. The hybrid catalyst shows high catalytic activity towards transamidation reaction at very low temperatures and in solvent-free conditions compared to pure ceria nanoparticles. The SiO_2_–CeO_2_ catalyst showed more than 99% selectivity and a remarkable catalytic activity of above 90% for the conversion of *N*-heptyl amine with acetamide to *N*-heptyl acetamide at a very low temperature of 120 °C for 3 hours. Furthermore, the catalyst remains stable and active for repeated catalytic cycles. It established 80% catalytic activity even after 4 repeated cycles making it suitable for multiple-time usages.

## Abstract 2

Mechanism of transamidation reaction between acetamide and *N*-heptyl amine to yield *N*-heptyl acetamide, catalyzed by mesoporous SiO_2_–CeO_2_ hybrid nanostructures.

## Graphical abstract

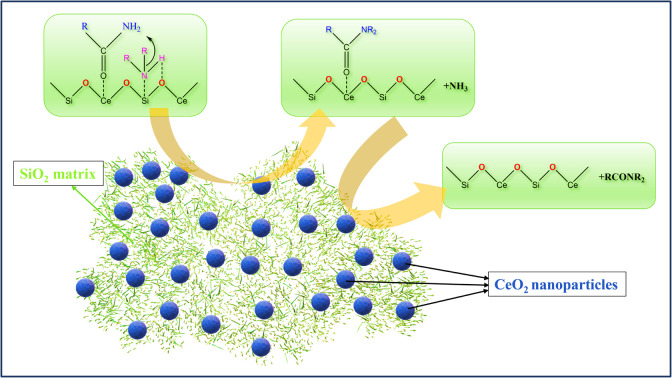

## 1. Introduction

Silica-supported metal oxides and noble metal active nanocomposites have been reported to improve the catalytic performance in various organic reactions.^1–5^ Ceria (CeO_2_) and ceria-based nanocomposite were used for reactions, such as carbon monoxide (CO) and hydrocarbon oxidation, fuel cell applications, and steam reforming with high catalytic activity.^6–11^ CeO_2_ was mainly employed as an acid–base catalyst for several organic reactions such as the alkylation of aromatic compounds, dehydration of alcohols, dimerization of aldehydes, alcohols, carboxylic acids, and esters to ketones, reduction of benzoic acid and the cyclization of diols.^12–16^ Mainly ceria-based noble metal catalysts were applied for different catalytic applications because of their high reactivity.^17–19^ From a green chemistry point of view, ceria has been receiving a lot of interest as a promoter, automobile exhauster, and pollution abatement material.^20–22^ Ceria is widely used in organic catalytic reactions because of its distinctive ability to transfer more oxygen and the possibility of variation of oxidation state between reduced and oxidized species (Ce^3+^ to Ce^4+^).^23–25^ Silica (SiO_2_) is also an extensively studied porous material having outstanding properties like high-temperature stability, larger surface area, and excessive absorption capacity, all of which are important factors for catalysis.^26–28^ Silica is naturally transparent, and ceria's lattice parameter (0.541 nm) closely resembles that of silica. Due to these properties, ceria was chosen for making composite with SiO_2_ nanoparticles.^29,30^ Silica-based ceria catalyst is one of the most effective metal oxide nanocomposites which allows a high catalyst loading, high activity and is cheaper than noble metals.^31,32^ Researchers have reported an increase in the activity of the catalysts by forming a composite with suitable support material.^33–35^ Al_2_O_3_, SiO_2_, TiO_2_, Fe_2_O_3_, MnO_2_, CeO_2_, and MgO are some of the key metal oxides used as a support due to their active redox properties, inertness, high thermal and mechanical stability.^36,37^ The inertness of CeO_2_ and SiO_2_ as well as the Au nanoparticle structure in Au/CeO_2_ and Au/SiO_2_ catalysts is mostly controlled by CeO_2_ and SiO_2_ structures' respectively, which play a crucial role in the catalytic action for oxidation of CO.^38,39^ CeO_2_ nanoparticles with metal oxides such as CeO_2_–ZrO_2_, CeO_2_–PbO_2_, CeO_2_–CuO, CeO_2_–MnO_*x*_, CeO_2_–TiO_2_, CeO_2_–Al_2_O_3_, and CeO_2_–SiO_2_ are widely used in catalyzed organic reactions.^40–46^ The applications of metal oxides in heterogeneous catalysis were proven to be highly efficient. For instance, palladium supported on CeO_2_ and ZnO synthesized by the co-precipitation method was reported to advance CO_2_ hydrogenation to yield formic acid.^47^ In a recent report, single-atom catalysts supported on metal oxo clusters were efficiently utilized for hydrodeoxygenation of biomass products as well as for alcohol and CO oxidation.^48^ Recently, it has been shown that the ordered SiO_2_ matrix in the composite not only improves the textural stability of CeO_2_ but also leads to a high surface area.^49^

Amides are one of the most essential functional groups, which are widely found in natural products, pharmaceuticals, and other materials.^50^ Thus, it is important to synthesize *N*-alkyl amides using efficient and cost-effective technology to develop different amide products. Earlier, in alkyl amide formation, amines directly react with amides and these reactions were mostly catalyzed by boronic acid derivatives, metal catalysts, and zirconocene dichloride.^51^ Usually, transamidation reactions proceed under high temperatures. Here, we designed a mesoporous silica–ceria nanocatalyst that has a high surface area, good dispersity, and high catalytic activity for transamidation reactions in solvent-free conditions at very low temperatures. The presence of silica as a support material for ceria nanoparticles plays an important role in enhancing the overall catalytic activity of the catalyst. Furthermore, this study sheds new light on the mechanisms behind the silica–ceria hybrid catalytic process.

## 2. Experimental

### 2.1 Materials

Analytical-grade chemicals were utilized throughout, with no further purification. Cerium nitrate (Sigma-Aldrich), tetraethyl orthosilicate (TEOS) (Alfa Aesar), cetyltrimethylammonium bromide (CTAB) (Spectrochem Laboratories), ethanol (Merck KGaA), and ammonia solution (Fisher Scientific) were used. Deionized water was used to make each solution.

### 2.2 Synthesis of mesoporous SiO_2_–CeO_2_ nanocomposite

Mesoporous SiO_2_–CeO_2_ catalyst was synthesized by the hydrolysis of cerium nitrate and tetraethyl orthosilicate in a water–ethanol system using the solvothermal method. In brief, about 0.5 g of cetyltrimethylammonium bromide and 1 M of tetraethyl orthosilicate (0.25 ml) were dissolved in a water–ethanol solution (7 : 6.3 ml). This mixture was stirred continuously for 30 min and 0.1 M cerium nitrate (0.58 g) was introduced to the reaction mixture and mixed well for 10 min. This was followed by dropwise adding 0.5 ml of 25% ammonia solution with continuous stirring. Finally, the reaction mixture was poured into a Teflon-lined autoclave and kept at 120 °C in a hot air oven for 48 h. The contents were then centrifuged and washed with distilled water and ethanol before being dried at 70 °C overnight. Pure ceria as well as silica nanoparticles were also synthesized by using similar solvothermal conditions. All the samples were calcined at 300 °C.

### 2.3 Catalytic experiments

For the transamidation of acetamide with *N*-heptyl amine, 25 mg of mesoporous SiO_2_–CeO_2_ (5.8 mol% Ce with respect to acetamide) was put into a blend of acetamide (2.5 mmol) and *N*-heptyl amine (5.0 mmol) in a round bottom flask attached to a condenser under N_2_ atmosphere and vigorously stirred at 120 °C for 3 h. After stirring, the SiO_2_–CeO_2_ catalyst was separated by centrifugation and washed using acetone four times. During the recycling experiment of the catalyst, it was calcined at 300 °C for 1 h. The resulting liquid products were identified by LC-MS. Conversion and selectivity were estimated based on acetamide and *N*-heptyl acetamide concentration in the mass spectra of the catalyst.

### 2.4 Characterization

A Bruker D8-Advance powder X-ray diffractometer with Cu-Kα radiation (*λ* = 1.5418 Å) was used to conduct the Powder X-ray Diffraction (PXRD) investigations. A Nicolet Protege 460 Fourier transform infrared (FT-IR) spectrometer was used to conduct FT-IR spectroscopy measurements utilizing KBr as the standard of transmission within the 400–4000 cm^−1^ range. With the FEI Tecnai G^2^ 20 electron microscope running at a 200 kV accelerating voltage, studies using transmission electron microscopy (TEM) as well as energy dispersive X-ray analysis (EDX) were conducted. Employing FEI Quanta 3D FEG/FESEM at an accelerating voltage of 20 kV, field emission scanning electron microscopy (FESEM) was conducted utilizing gold-coated discs. Using barium sulphate as a reference, the diffuse reflectance spectra of the samples were captured using the UV-Visible spectrophotometer Shimadzu UV-2450 in the wavelength range of 200–800 nm. Using a Nova 2000e Surface Area and Pore Size Analyzer (Quantachrome Instruments), nitrogen adsorption–desorption isotherms were recorded at liquid nitrogen temperature (77 K), and the specific area was calculated using the Brunauer–Emmett–Teller (BET) technique. Before the surface area measurements, the samples were degassed for 12 hours at 150 °C.

## 3. Results and discussion

**Fig. 1:**
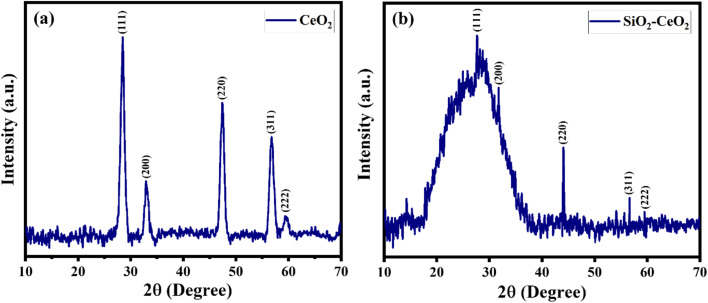
PXRD pattern of (a) CeO_2_ nanoparticles, (b) SiO_2_–CeO_2_ nanocomposite.

The powder X-ray diffraction patterns of pure ceria nanoparticles are displayed in Fig. 1(a), displaying peaks corresponding to the cubic phase of CeO_2_ which matched well with JCPDS card no. 81–0792. The PXRD pattern of the SiO_2_–CeO_2_ catalyst reveals the notable diffuse peak of silica over the angular range of 15° to 35° indicating the amorphous nature of the material and showing the intensity peaks of ceria nanoparticles confirming the formation of the composite (Fig. 1(b)). Table 1 summarizes the infrared band assignments. The weak band at 1635 cm^−1^ (water bending) and the Si–O–Si asymmetric stretching band at 1006 cm^−1^ both served as indicators of the presence of SiO_2_. At around 464 cm^−1^, the Si–O–Si asymmetric bending band was seen.^52^ The metal–oxygen band of ceria is responsible for the FT-IR band seen at around 1384 cm^−1^.^53^

**Table 1:** FT-IR band assignment of SiO_2_–CeO_2_ nanocomposite

Band position (cm^−1^) ceria	Band position (cm^−1^) silica	Band position (cm^−1^) silica–ceria	Band assignment
3412	3435	3400	–OH stretching
1635	1635	1631	H–O–H bending
—	802	1006	Si–O–Si stretching
—	467	464	Si–O–Si bending
851, 1383	—	1384	Ce–O–Ce

**Fig. 2:**
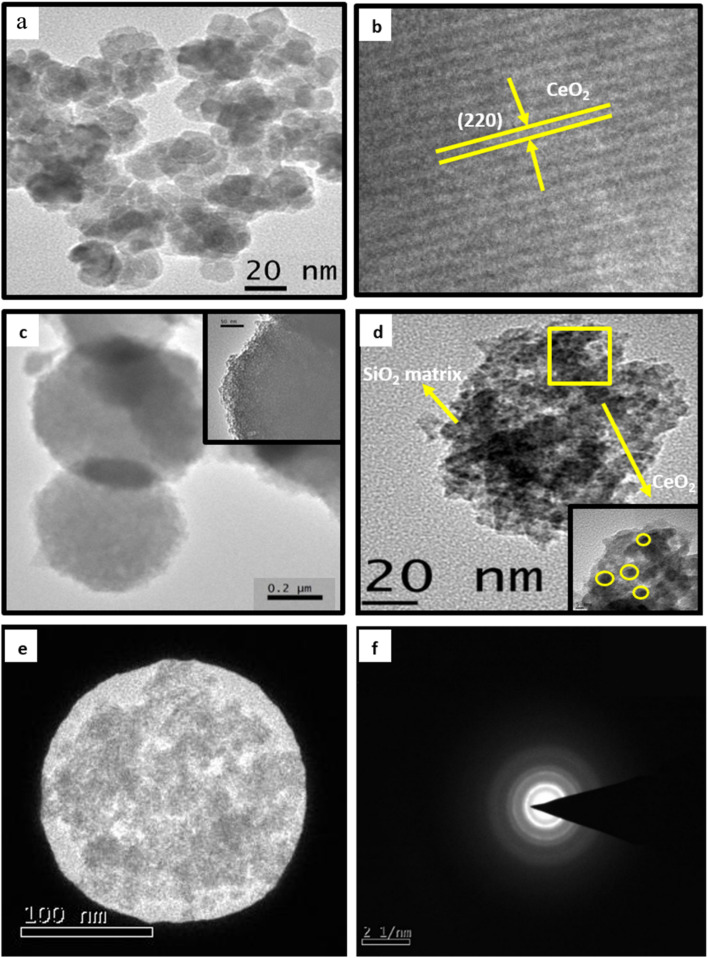
(a) TEM and (b) HRTEM images of CeO_2_ nanoparticles (c) TEM image of SiO_2_ spheres (d) TEM images of SiO_2_–CeO_2_ nanocomposite (e) and (f) selected area electron diffraction pattern of SiO_2_–CeO_2_ nanocomposite.

TEM and HRTEM images of ceria nanoparticles are shown in Fig. 2(a) and (b). A sphere-like morphology can be seen in the TEM image of pure ceria nanoparticles. Ceria nanoparticles were found to have an average particle size of 20 to 30 nm. HRTEM image of ceria nanoparticles shows the reflection corresponding to the plane of (220) cubic structure of ceria. Pure silica particles show spheres with an average particle size of 0.5 to 0.7 μm (Fig. 2(c)). TEM images of SiO_2_–CeO_2_ nanocomposite show the mesoporous nanostructure where spherical-shaped ceria nanoparticles are dispersed inside the silica matrix (Fig. 2(d)). The TEM image showed that the ceria nanoparticles in the composite material had an average particle size of about 5 nm. Fig. 2(e) and (f) exhibit the selected area electron diffraction pattern of SiO_2_–CeO_2_ nanocomposite which shows feeble concentric rings and the less crystalline nature of the material.

**Fig. 3:**
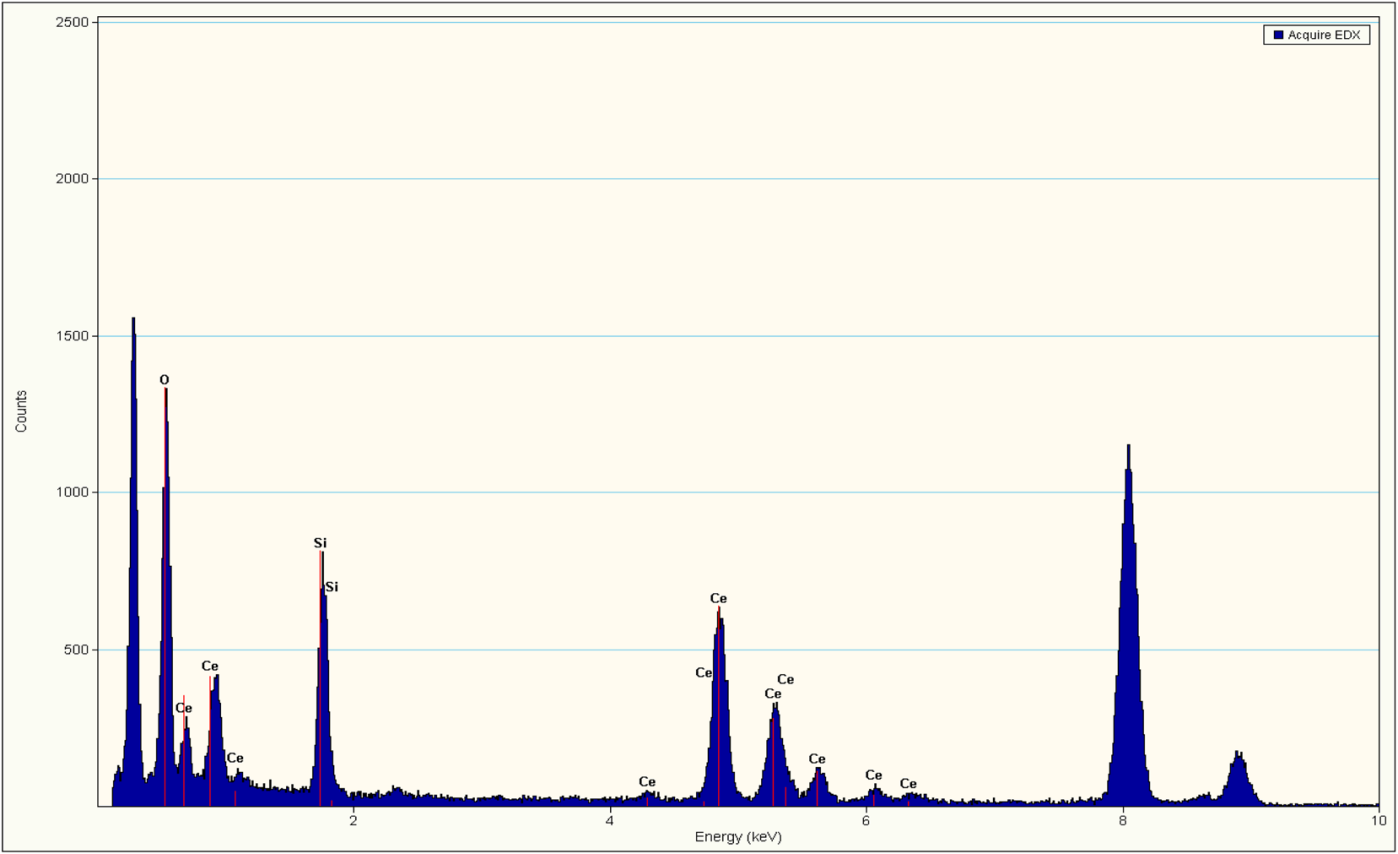
EDX data of SiO_2_–CeO_2_ nanocomposite.

**Fig. 4:**
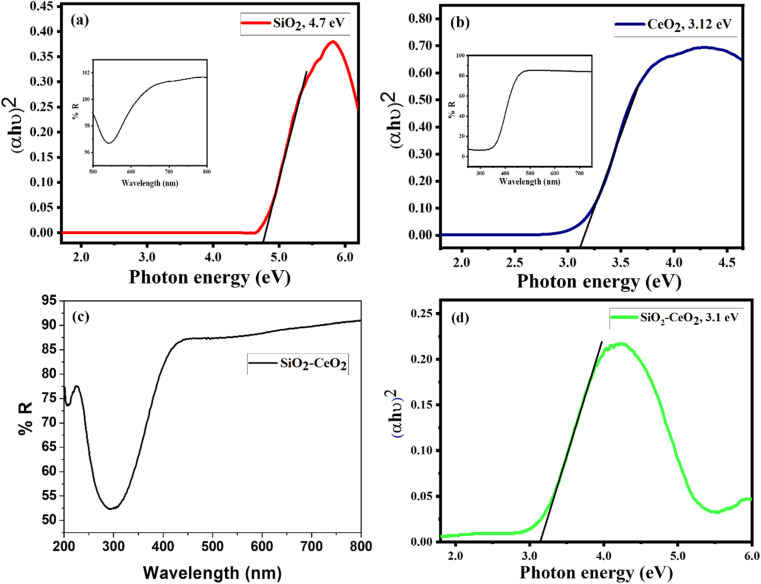
Tauc plot showing optical band gap of (a) SiO_2_ (b) CeO_2_ with diffuse reflectance spectra of SiO_2_ and CeO_2_ in their respective insets. (c) Diffuse reflectance spectra of SiO_2_–CeO_2_ nanocomposite. (d) Tauc plot showing optical band gap of SiO_2_–CeO_2_ nanocomposite.

The presence of Ce, O, and Si in silica–ceria catalyst was also confirmed by the EDX data of composite materials (Fig. 3). Diffuse reflectance spectroscopy (DRS) in the 200 to 800 nm range has been used to investigate the optical characteristics and to obtain band gap of the mesoporous catalyst (Fig. 4(c)). DRS spectra of silica–ceria catalyst shows reflectance at the wavelength of 300 nm. The Kubelka–Munk equation was applied for the band gap calculation using reflectance values and the calculated band gap of the mesoporous catalyst was 3.1 eV (Fig. 4).^54,55^ The band gap of pure silica and ceria nanoparticles were found to be 4.7 eV and 3.12 eV respectively. The silica–ceria composite band gap is very close to the band of ceria in UV range because of the effect of ceria presence in sample while the effect of silica in band gap is not significantly observed may be due to the wide bandgap of the silica.

**Fig. 5:**
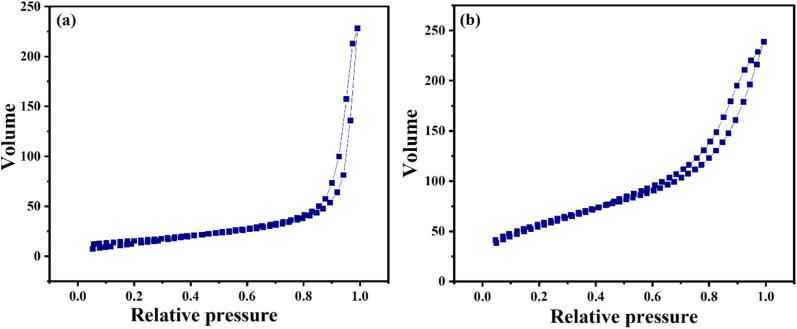
Absorption desorption isotherm of (a) CeO_2_ nanoparticles (b) SiO_2_–CeO_2_ nanocomposite.

The adsorption–desorption plot of pure CeO_2_ and SiO_2_–CeO_2_ nanocomposite is presented in Fig. 5(a) and (b) respectively. The samples are demonstrating an H-type hysteresis loop and according to IUPAC classification, the nitrogen adsorption–desorption isotherm of CeO_2_ and SiO_2_–CeO_2_ nanocomposite is of type IV indicating their mesoporous nature.^56^

The pore volume, pore size, and surface area of silica, ceria, and mesoporous silica–ceria nanocomposites have been shown in Table 2. The synthesized silica–ceria catalyst's specific surface area was observed to be 206 m^2^ g^−1^ which was much higher in comparison to pure silica spheres (86 m^2^ g^−1^) as well as ceria nanoparticles (55 m^2^ g^−1^). The high surface area, high absorption capacity, and acidic nature of silica support enhance the catalytic activity of mesoporous SiO_2_–CeO_2_ nanocomposite. The Lewis acidity of silica and the acid–basic nature of ceria nanoparticles together plays a significant role to improve the catalytic activity of the SiO_2_–CeO_2_ nanocomposite.^57,58^

**Table 2:** Total pore volume, average pore size, and surface area of SiO_2_ spheres, CeO_2_ nanoparticles, and SiO_2_–CeO_2_ nanocomposite

Sample	Total pore volume (cm^3^ g^−1^)	Average pore size (nm)	Surface area (m^2^ g^−1^)
SiO_2_	0.16	15	86
CeO_2_	0.36	20	55
SiO_2_–CeO_2_	0.37	15	206

The mesoporous nature and high surface area of the material increase the loading and absorption capacity of the material and influence the catalytic activity of the catalyst.^59,60^ The pore volume of SiO_2_–CeO_2_ nanocomposite material was observed as 0.37 cm^3^ g^−1^ while the pore volume of pure silica and ceria was observed as 0.16 cm^3^ g^−1^ and 0.36 cm^3^ g^−1^ respectively. The pore size of silica, ceria, and silica–ceria composite on average was obtained to be 15 nm, 20 nm, and 15 nm respectively. The surface area of silica, ceria, and silica–ceria composite was found to be 86 m^2^ g^−1^, 55 m^2^ g^−1^, and 206 m^2^ g^−1^ respectively (Table 2).

### 3.1 Catalytic activity of silica–ceria mesoporous nanocomposite

**Fig. 6:**
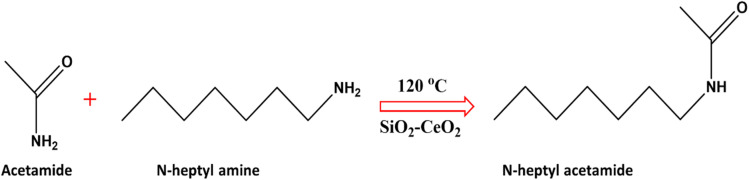
Transamidation reaction catalyzed by SiO_2_–CeO_2_ nanocomposite.

Transamidation reaction was chosen as a model reaction to study the synthesized SiO_2_–CeO_2_ nanocomposite's catalytic performance (Fig. 6). Pure silica and pure ceria nanoparticles were also used to compare the effectiveness of the nanocomposite. The catalytic activity of the SiO_2_–CeO_2_ nanocomposite for transamidation reactions was studied using *N*-heptyl amine in presence of acetamide. 50 mg of catalyst was used in each reaction at different temperatures of 120 °C, 150 °C, and 180 °C and varying the period of 30 min, 1 h, 2 h, and 3 h. From LC-MS studies, it was observed that pure silica and ceria catalysts show low catalytic activity as compared to silica–ceria nanocomposite. The catalytic activity of pure ceria catalyst in the presence of *N*-heptyl amine and acetamide was observed to be ∼15%. When SiO_2_ was used as a catalyst, no activity was found with *N*-heptyl amine. From the LC-MS results, it was confirmed that the SiO_2_–CeO_2_ catalyst was very effective for transamidation reaction. This SiO_2_–CeO_2_ catalyst is reported for the first time for use in transamidation reaction and it shows high selectivity and remarkable catalytic activity (>90%) with *N*-heptyl amine and acetamide at an optimal temperature of 120 °C temperature and for 3 h. Table 3 shows the catalytic activity (% conversion) of reactants under different reaction conditions.

**Table 3:** Catalytic activity and % conversion of reactants in the presence of SiO_2_, CeO_2,_ and SiO_2_–CeO_2_ nanoparticles as catalysts

Catalyst	Amide	Amine	Temperature	Time	% conversion	Selectivity
CeO_2_	Acetamide	*N*-Heptyl amine	120 °C	2 h	0	0
CeO_2_	Acetamide	*N*-Heptyl amine	170 °C	2 h	15	>99
Com CeO_2_	Acetamide	*N*-Heptyl amine	120 °C	2 h	0	0
Com CeO_2_	Acetamide	*N*-Heptyl amine	150 °C	2 h	0	0
Com CeO_2_	Acetamide	*N*-Heptyl amine	170 °C	2 h	0	0
SiO_2_	Acetamide	*N*-Heptyl amine	120 °C	2 h	0	0
SiO_2_	Acetamide	*N*-Heptyl amine	150 °C	2 h	0	0
SiO_2_	Acetamide	*N*-Heptyl amine	170 °C	2 h	0	0
SiO_2_–CeO_2_	Acetamide	*N*-Heptyl amine	120 °C	30 min	11	>99
SiO_2_–CeO_2_	Acetamide	*N*-Heptyl amine	120 °C	1 h	51	>99
SiO_2_–CeO_2_	Acetamide	*N*-Heptyl amine	120 °C	2 h	64	>99
SiO_2_–CeO_2_	Acetamide	*N*-Heptyl amine	120 °C	3 h	90	>99
SiO_2_–CeO_2_	Acetamide	*N*-Heptyl amine	120 °C	5 h	90	>99

**Fig. 7:**
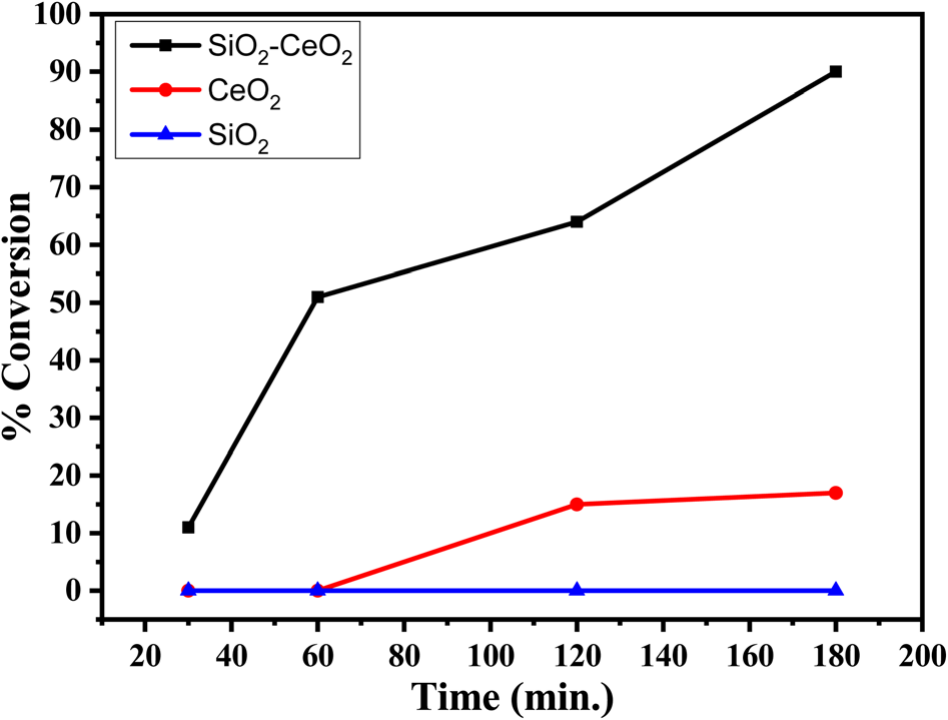
Percentage conversion *versus* time plot with SiO_2_, CeO_2_, and SiO_2_–CeO_2_ catalysts.

**Fig. 8:**
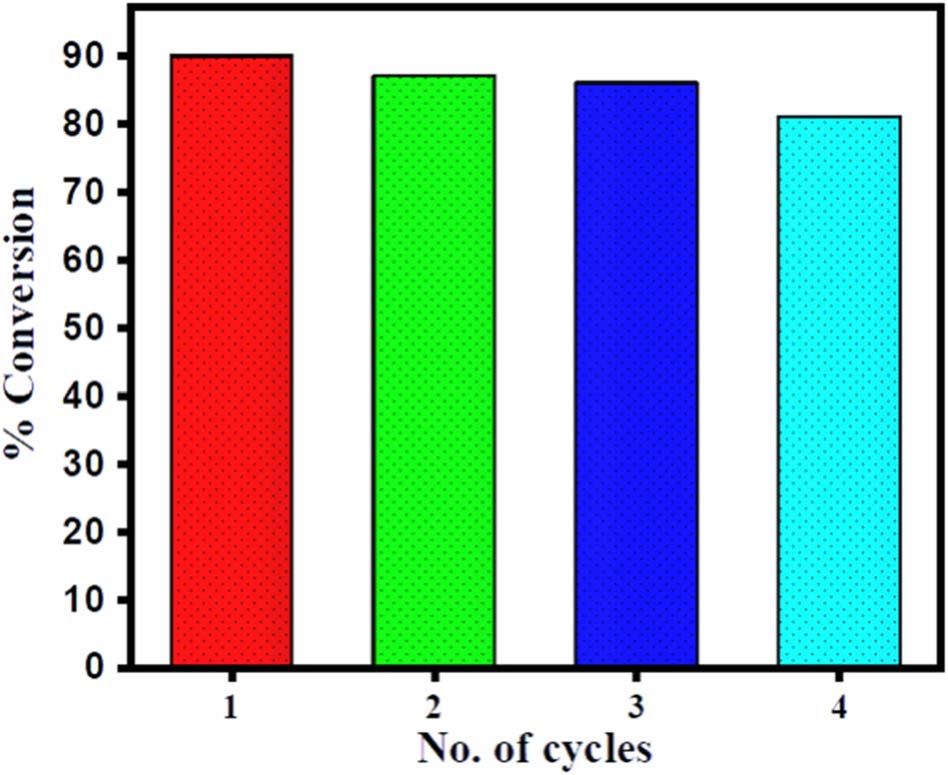
Recyclability of SiO_2_–CeO_2_ nanocomposite as a catalyst.

The % conversion *versus* time plot of SiO_2_–CeO_2_, CeO_2_, and SiO_2_ is presented in Fig. 7. The conversion was found to be greater than 90% in the presence of the composite, whereas the CeO_2_ catalyst shows only ∼20% conversion and there was no conversion in presence of SiO_2_. The recyclability of the SiO_2_–CeO_2_ catalyst is shown in Fig. 8. The catalyst was calcined at 300 °C for 1 h before each recycling study. Reactions using the SiO_2_–CeO_2_ catalyst show ∼80% conversion after four consecutive cycles which show that the composite catalyst is stable and active even after four catalytic cycles and thus can be reused multiple times. The XRD pattern of the recycled catalyst after 4 cycles are presented in Fig. S10,† which shows all the peaks present in the fresh catalyst with reduced intensity, establishing the stability of the catalyst.

### 3.2 Mechanism

**Scheme 1:**
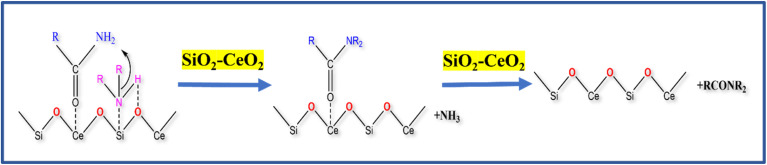
Reaction mechanism of SiO_2_–CeO_2_ catalyst.

The CeO_2_ nanoparticles on silica matrix with a high surface area and good absorption capacity accelerate its catalytic activity. It is reported in the literature that CeO_2_ nanoparticles have acid–base pair active sites.^61,62^ The probable reaction mechanism of transamidation reaction using SiO_2_–CeO_2_ catalyst is shown in Scheme 1. In the first step of the reaction, the activation of acetamide at the acid site of ceria is possible in the composite. At the same time, the heptyl amine species (basic) coordinates with acidic (NH–Ce) and basic (H–O) sites of ceria. The addition of primary amine with acetamide gives the *N*-heptyl acetamide transition state. Finally, in step 3, *N*-heptyl acetamide is isolated from the acid–base site of the mesoporous SiO_2_–CeO_2_ catalyst. The high surface area of silica and the coexistence of acid–basic sites of ceria is important for the activation of both an acidic and a basic site to make transamidation of the amine simple. The proposed mechanism was supported by an FTIR *ex situ* pyridine absorption study to prove the presence of various Brønsted and Lewis acid sites on the catalyst surface.^63^ Fig. S6† shows the FTIR vibration band of the Brønsted acid site at 1541 cm^−1^ and the Lewis acid site band at 1456 cm^−1^. This indicates that the reaction proceeds by the influence of various acidic sites present on the catalyst surface.

## 4. Conclusions

The present study shows the high selectivity and catalytic activity (>90%) of the SiO_2_–CeO_2_ catalyst. The conversion of *N*-heptyl amine with acetamide in presence of SiO_2_–CeO_2_ catalyst is a facile and solvent-free route for the transamidation reaction. The high surface area, the absorption capacity of silica, and the acid–basic site of ceria are the main reason for the enhanced catalytic activity of SiO_2_–CeO_2_ nanocomposite. The prepared SiO_2_–CeO_2_ catalyst showed good catalytic activity (>80%) even after four consecutive catalytic cycles and proved that it is highly stable and retains its catalytic activity for multiple uses.

## Conflicts of interest

There are no conflicts to declare.

## Supplementary Material

RA-013-D3RA01552A-s001
