# Supplementary material for: Synthesis of mesoporous SiO2–CeO2 hybrid nanostructures with high catalytic activity for transamidation reaction
Source: RSC Adv. 2023 Apr 28;13(19):13134–41. doi: 10.1039/d3ra01552a (PMC10140673; doi:10.1039/d3ra01552a)
Supplement: RA-013-D3RA01552A-s001 [file RA-013-D3RA01552A-s001.pdf]

Electronic Supplementary Material (ESI) for RSC Advances.
This journal is © The Royal Society of Chemistry 2023

Synthesis of mesoporous SiO_2-CeO_2 hybrid nanostructures with high catalytic activity for transamidation reaction

Manu Sharma[a*], Harikrishnan K[a], Umesh Kumar Gaur[b*], Ashok K. Ganguli[c]

[a]*Central University of Gujarat, Gandhinagar, India*

[b]*VP & RPTP Science College, Vallabh Nagar, India*

[c]*Indian Institute of Technology Delhi, New Delhi, India*

[*]*Corresponding author*

FTIR spectra of CeO_2, SiO_2, and the nanocomposite of CeO_2 and SiO_2 are given in Fig. S1, S2, and S3 respectively. Fig. S1 shows the wide band around the region of 3412 cm^{-1} which is accredited to -OH stretching vibrations and the band position near 1635 cm^{-1} belongs to H-O-H bending of residual water. The peak around 851 cm^{-1} is due to the Ce-O-Ce stretching vibrations [1]. In Fig. S2, the broad band around 3435 cm^{-1} is due to -OH stretching vibrations. The signal at 1635 cm^{-1} is assigned to hydroxyl groups present at the surface of mesoporous silica [2]. The band corresponding to 802 cm^{-1} and 467 cm^{-1} were assigned to Si-O-Si symmetric stretching and bending vibrations respectively [3,4]. In Fig. S3, the plot of the SiO_2-CeO_2 composite shows the band position corresponding to 1384 cm^{-1} representing Ce-O-Ce vibrations. Also, the peaks at 1006 cm^{-1} and 464 cm^{-1} in the composite spectra represent the Si-O-Si stretching and bending vibrations respectively and confirm the presence of both silica and ceria in the composite.

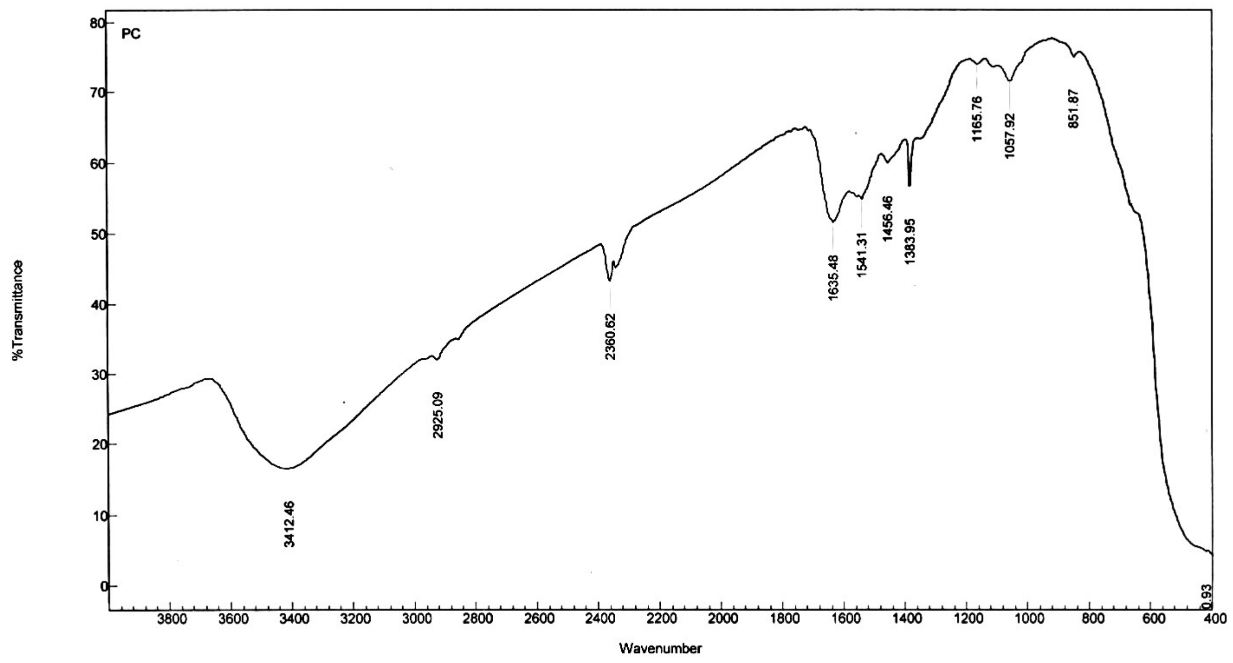

Fig. S1. : FTIR spectra of ceria nanoparticles

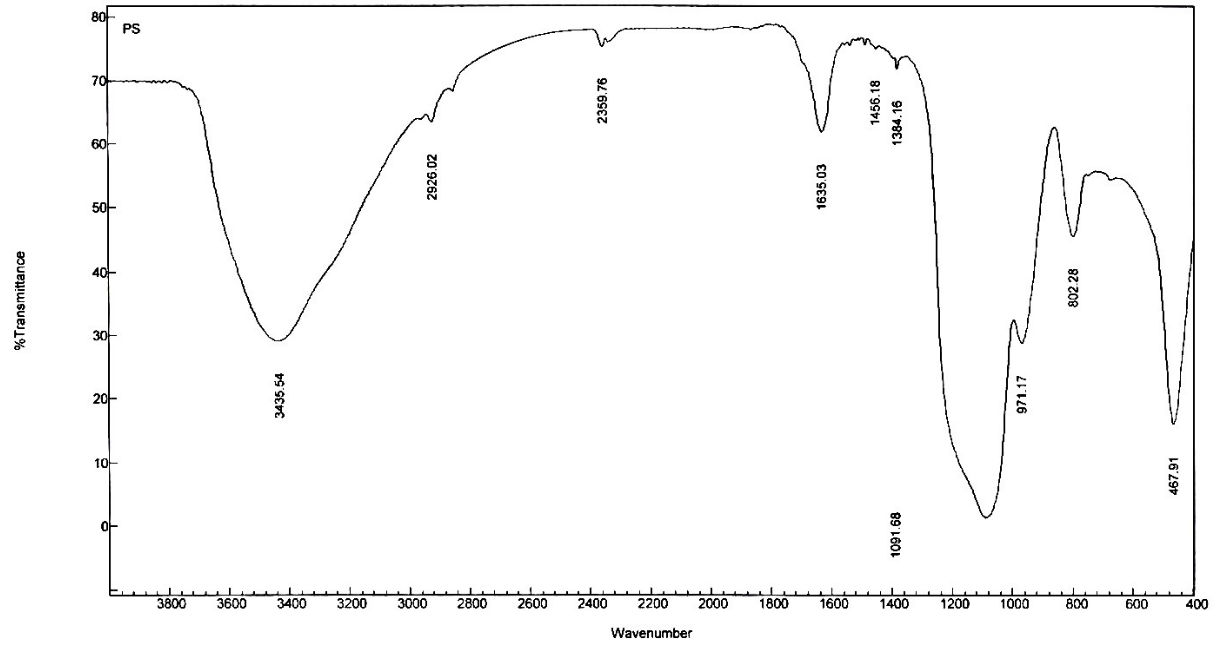

Fi

g. S2. : FTIR spectra of silica nanoparticles

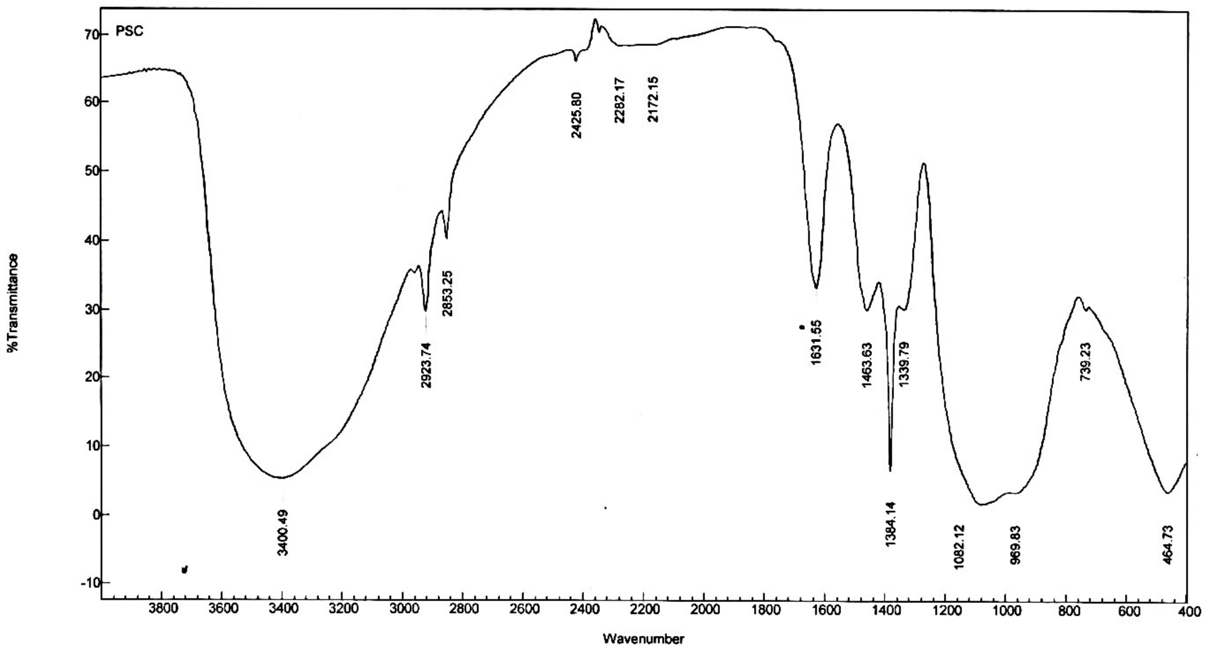

F

ig. S3. : FTIR spectra of Silica-Ceria nanoparticles

Fig. S4 shows the EDX result corresponding to ceria nanoparticles that confirm the presence of cerium and oxygen in the entire sample. EDX result of pure silica spheres confirms the presence of Si and O in the material as shown in Fig. S5.

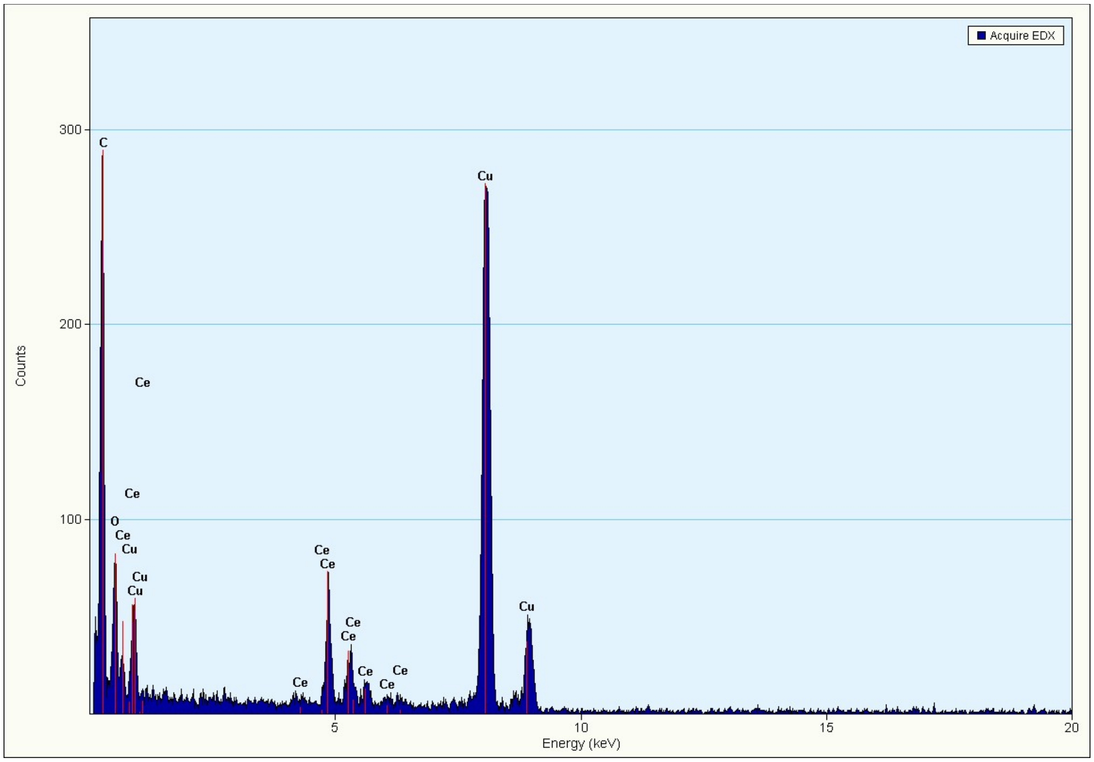

Fig. S4. : EDX data of CeO_2 nanoparticles.

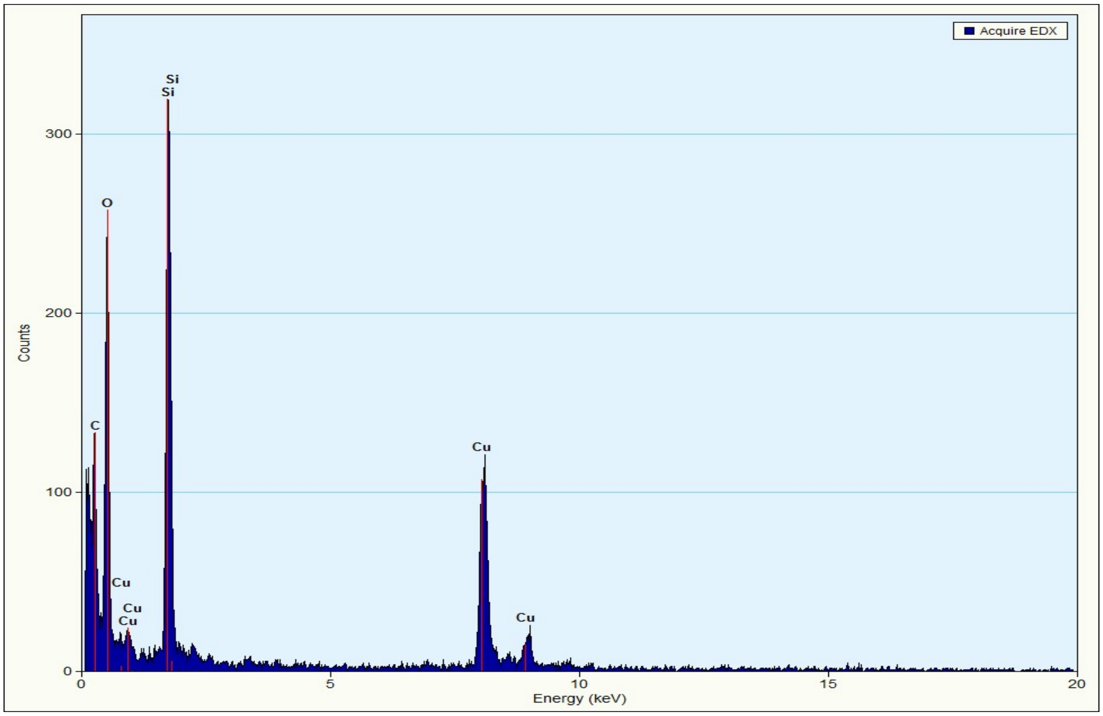

Fig. S5. : EDX data of SiO_2 spheres.

In Fig. S6, the FT-IR ex-situ pyridine absorption spectra of the SiO_2-CeO_2 catalyst show different acidic sites on the surface of the composite material. The FT-IR vibration band due to the Bronsted acid site appears at 1541 cm^{-1} whereas a band observed at 1456 cm^{-1} is due to the Lewis acid site of the catalyst. The pyridine absorption study indicates that the reaction proceeds in the influence of Lewis acidic site as well as Bronsted acidic site on the catalyst surface [5]

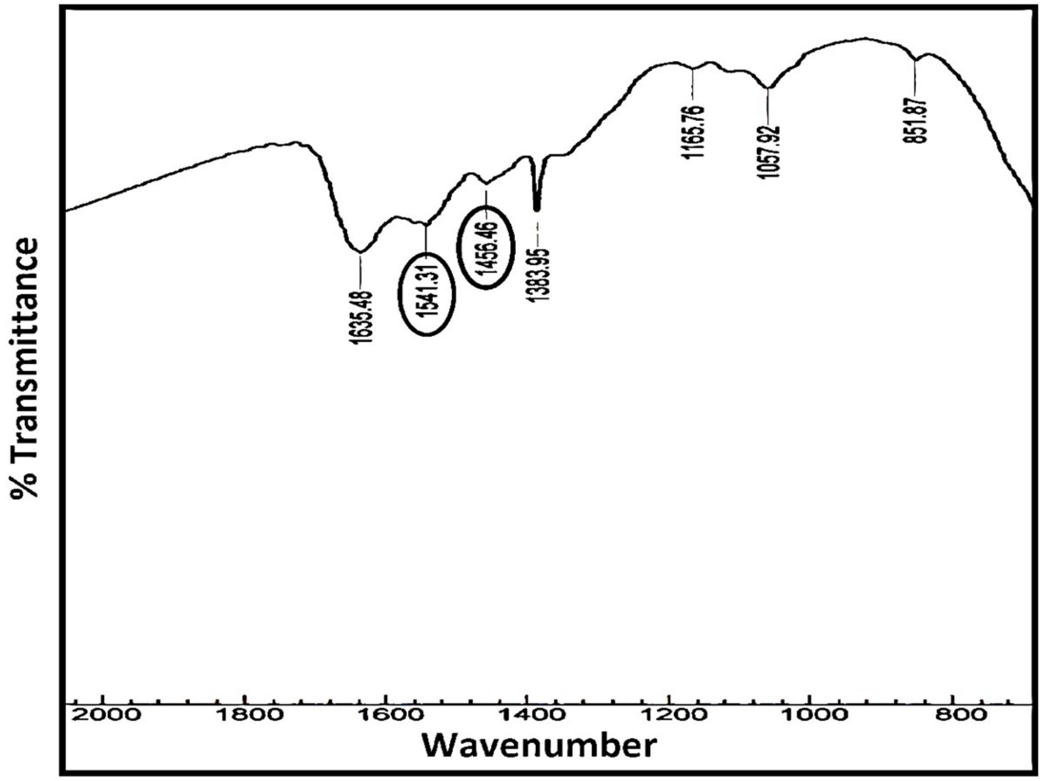

Fig. S6. : The FT-IR pyridine absorption spectra of the SiO_2-CeO_2 catalyst

The LC-MS analysis of liquid products obtained after catalysis using SiO_2-CeO_2 nanocomposite at 120 °C for 1 h, 2 h, and 3 h are shown in Fig. S7, S8, and S9 respectively. The peak obtained in the range of 156 to 158 m/z shows the formation of the product, N-heptyl acetamide after transamidation reaction catalyzed by SiO_2-CeO_2 nanocomposite at different reaction parameters provided.

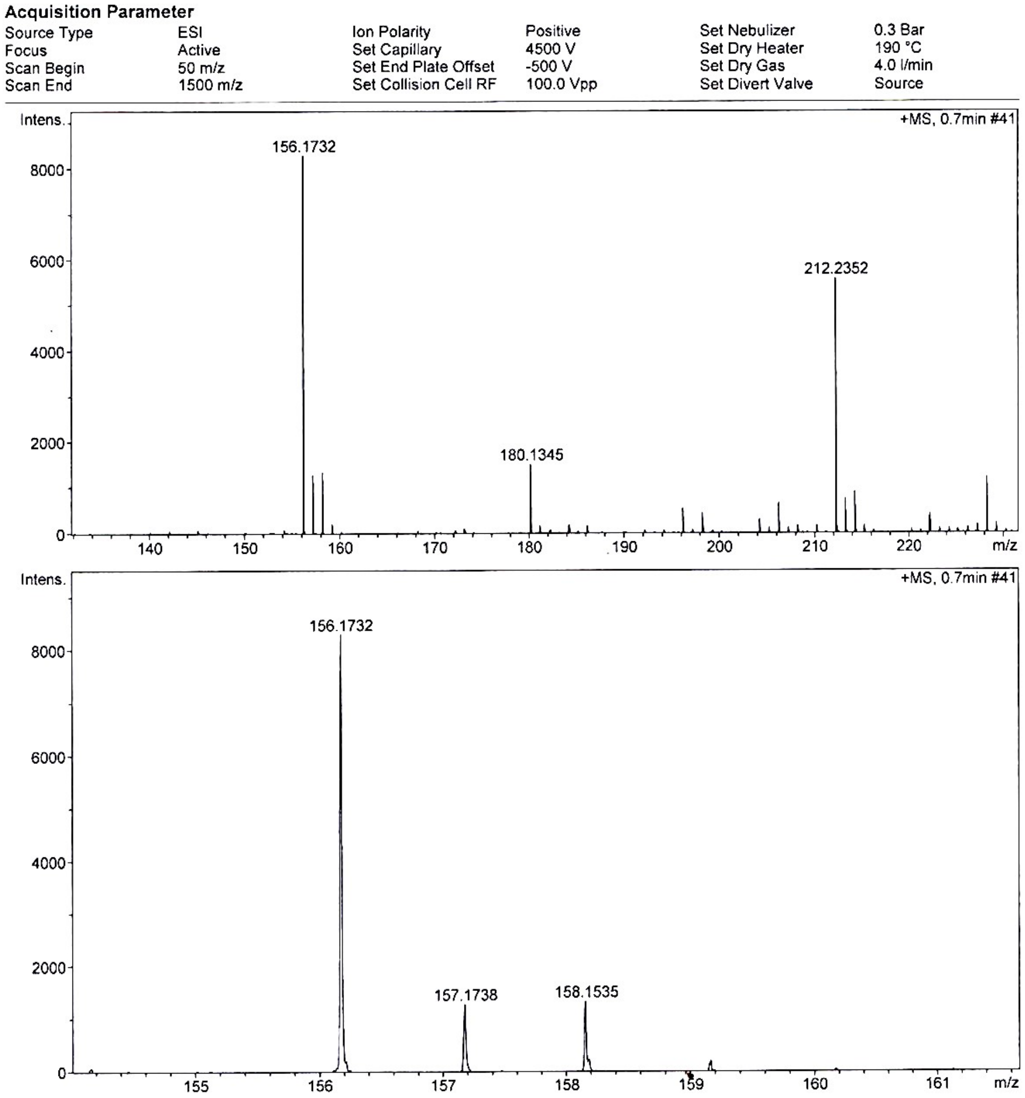

Fig. S7. : LC-MS analysis of liquid products obtained after catalysis using SiO_2-CeO_2 nanocomposite for 120 °C, 1 h.

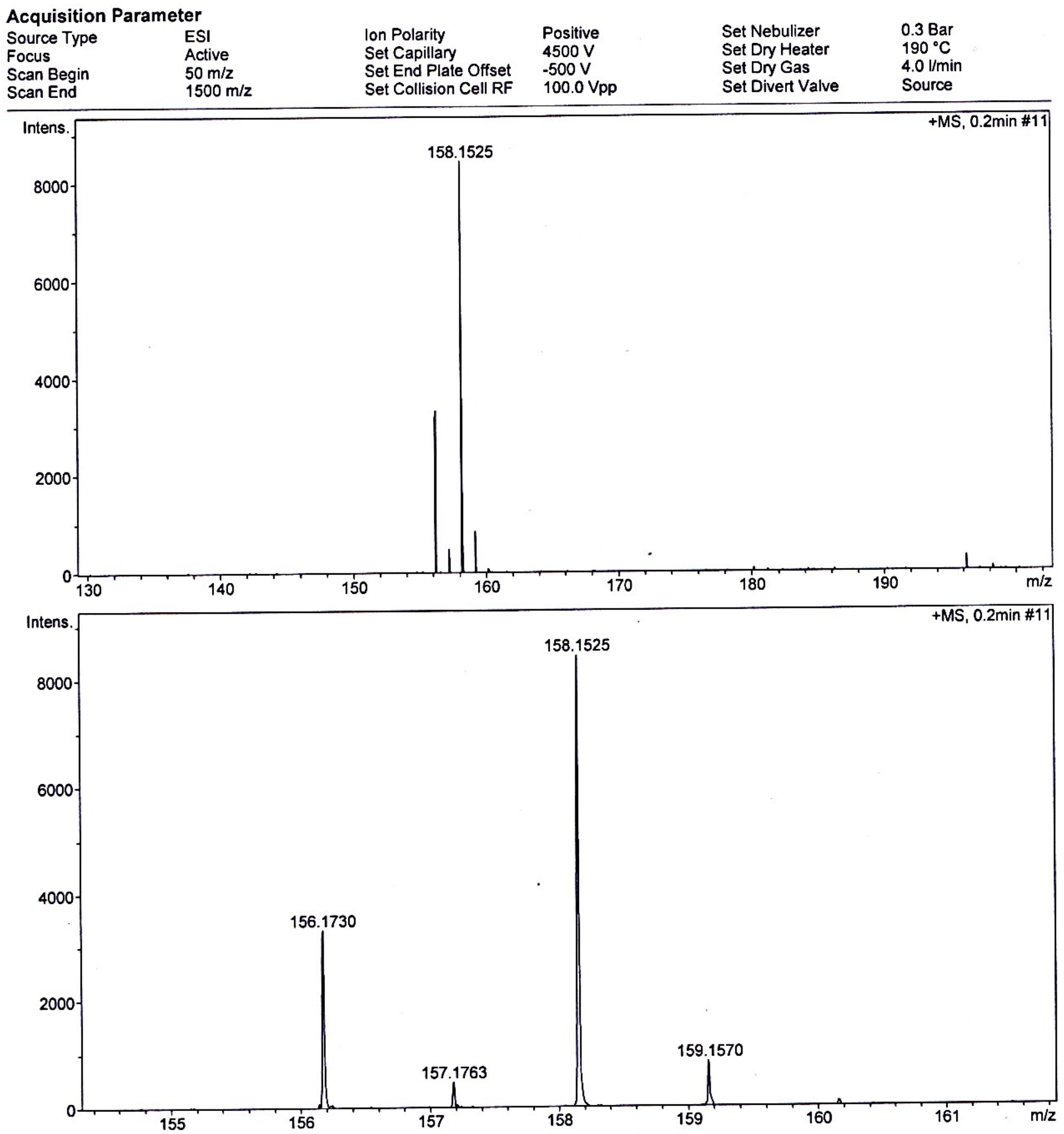

Fig. S8. : LC-MS analysis of liquid products obtained after catalysis using SiO_2-CeO_2 nanocomposite for 120 °C, 2 h.

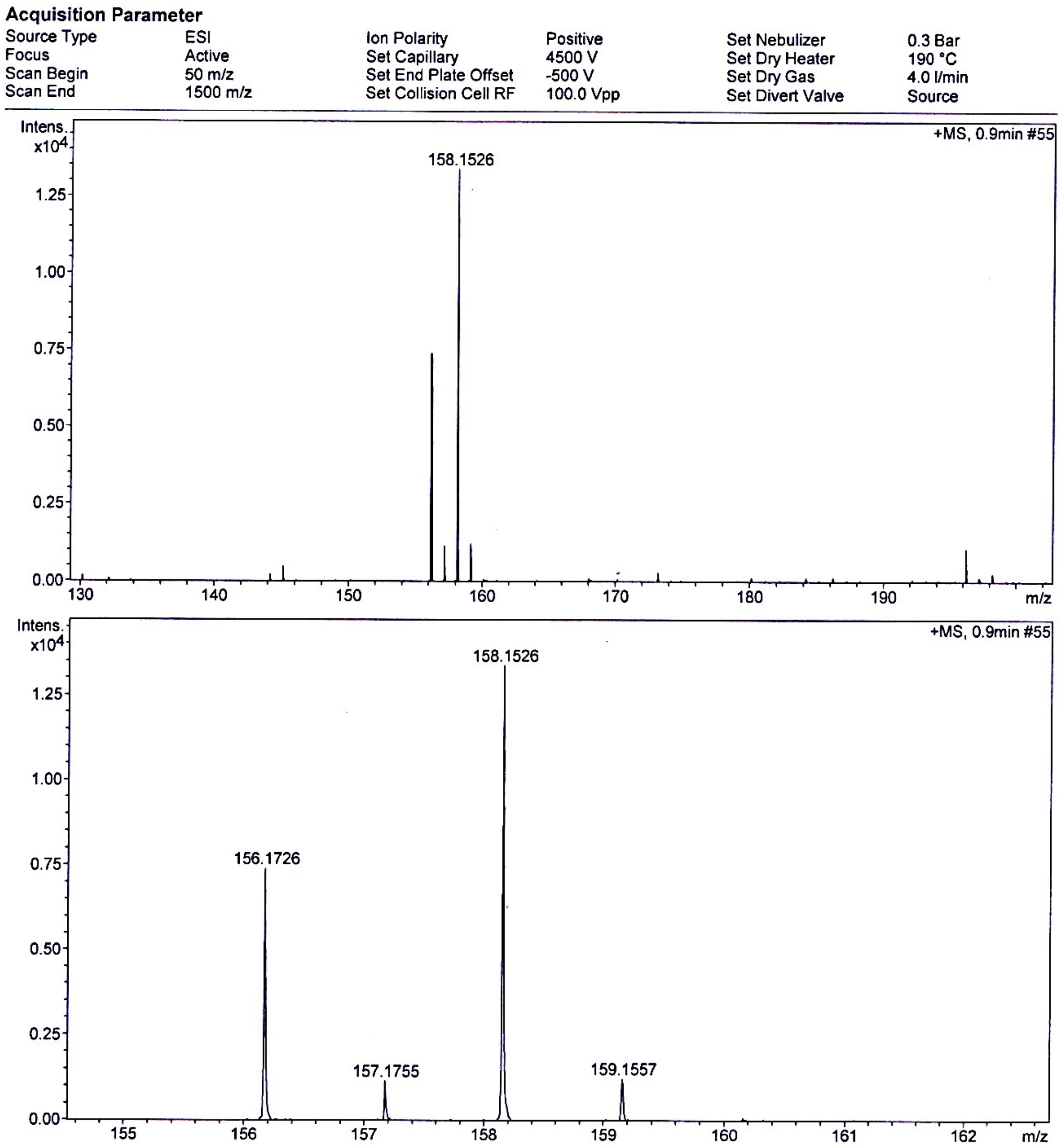

Fig. S9. : LC-MS analysis of liquid products obtained after catalysis using SiO_2-CeO_2 nanocomposite for 120 °C, 3 h.

The transamination reaction was also studied using N-hexyl amine in the presence of acetamide. 50 mg of catalyst was used in each reaction at a temperature of 150 °C for a 3 h period. When using SiO_2 and CeO_2 as catalysts, no activity or conversion was observed. Meanwhile, the catalytic

activity of SiO_2-CeO_2 catalyst in the presence of N-hexyl amine and acetamide was observed to be ~ 80 %. The results are presented in Table No S1.

Table S1. : Catalytic activity and % conversion of reactants in the presence of SiO_2, CeO_2, and SiO_2-CeO_2 nanoparticles as catalysts.

Catalyst	Amide	Amine	Temperature	Time	% Conversion	Selectivity
CeO_2	Acetamide	N-hexyl amine	150 °C	3 h	0	0
SiO_2	Acetamide	N-hexyl amine	150 °C	3 h	0	0
SiO_2-CeO_2	Acetamide	N-hexyl amine	150 °C	3 h	80	>99

The PXRD patterns of fresh SiO_2-CeO_2 catalyst and recycled SiO_2-CeO_2 catalyst after 4 catalytic cycles are presented in Fig. S10. The recycled catalyst was calcined at 300 °C before characterization using XRD. All the peaks present in the fresh catalyst were also present in the recycled catalyst with reduced intensities which indicates that the catalyst has undergone little changes during the catalytic process and is stable. Thus, the catalyst can be reused multiple times. TEM images of the recycled catalyst after 4 cycles are also shown in Fig. S11. Spherical-shaped ceria nanoparticles can be seen dispersed in the silica matrix indicating the stability of the catalyst.

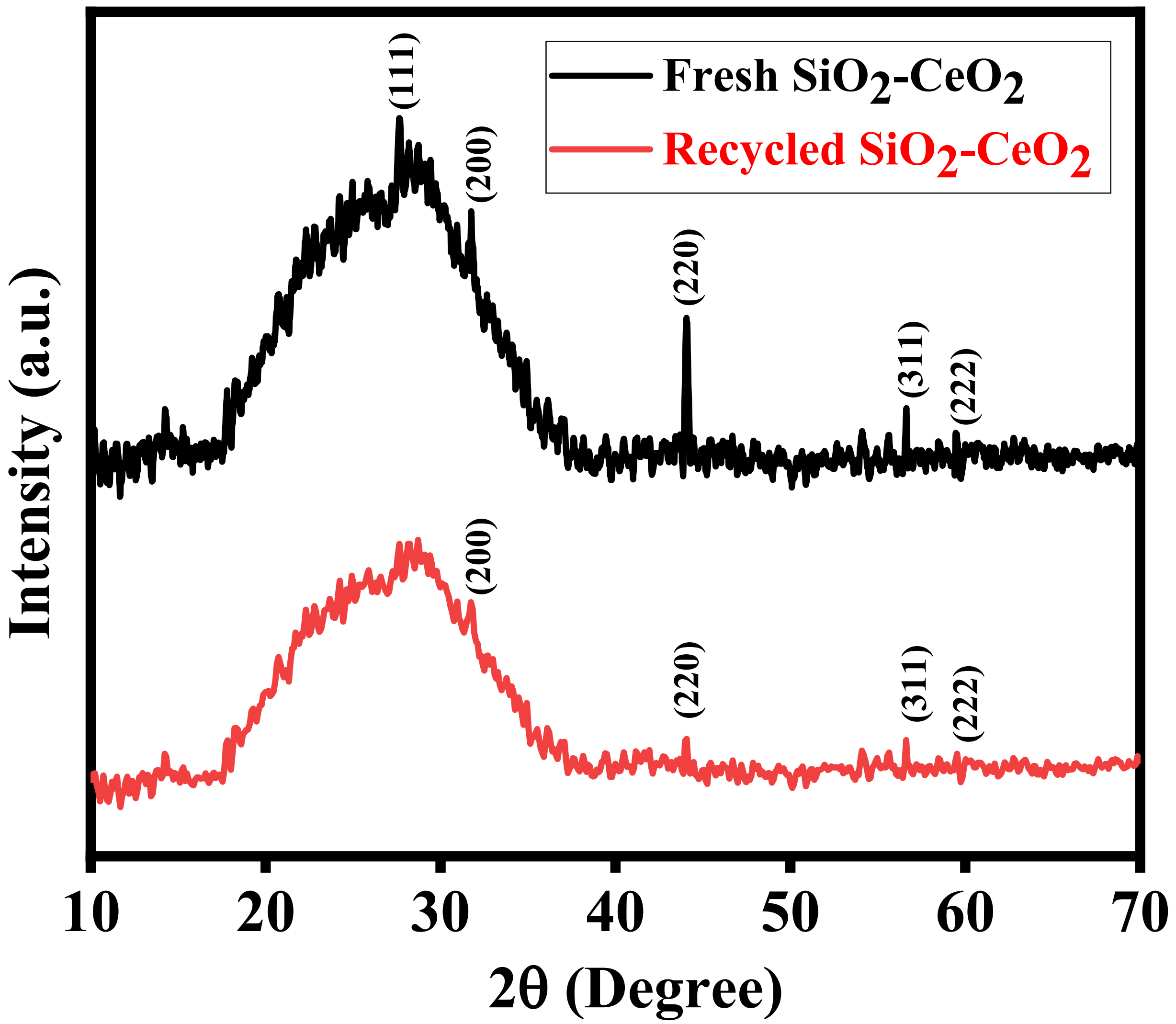

Fig. S10. : PXRD patterns of fresh SiO_2-CeO_2 nanocomposite and recycled SiO_2-CeO_2 nanocomposite after 4 cycles.

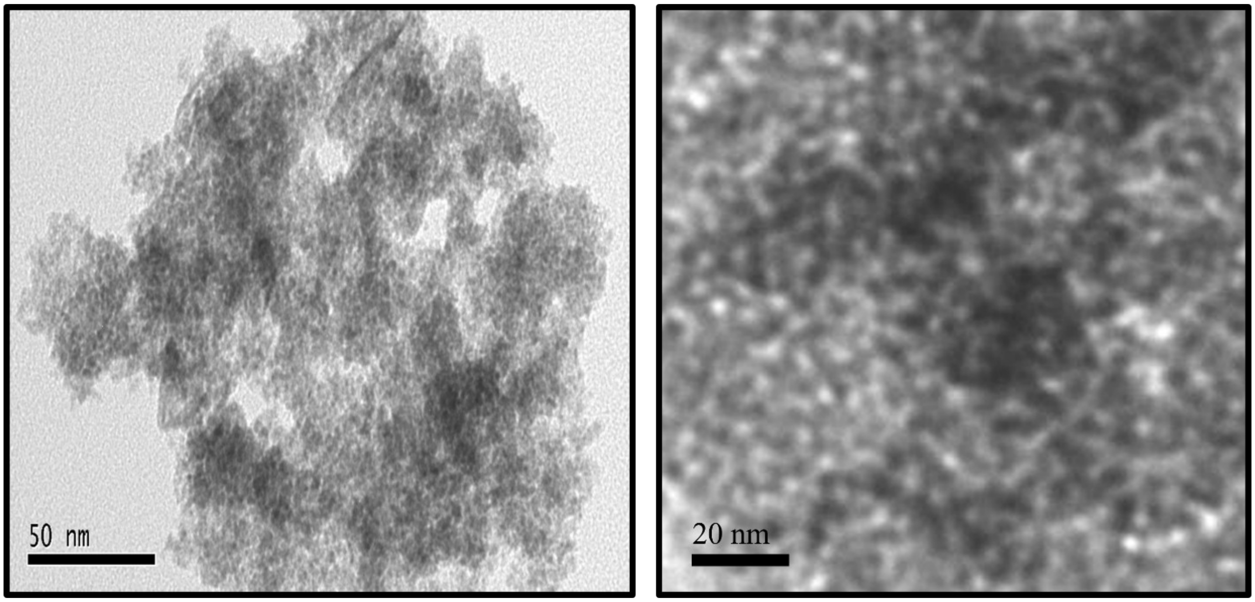

Fig S11. : TEM image of SiO2-CeO2 catalyst after 4 catalytic cycles

References

1 A. S. Fudala, W. M. Salih and F. F. Alkazaz, *Materials Today: Proceedings*, 2022, **49**, 2786–2792.

2 S. Mirfakhraee, R. Bafkary, Y. H. Ardakani and R. Dinarvand, *Journal of Nanoparticle Research*, 2022, **24**, 100.

3 E. Salimi and A. K. Nigje, *Carbohydrate Polymers*, 2022, **298**, 120077.

4 X. Wen, *RSC Advances*, 2019, **9**, 13908–13915.

5 V. Zholobenko, C. Freitas, M. Jendrlin, P. Bazin, A. Travert and F. Thibault-Starzyk, *Journal of Catalysis*, 2020, **385**, 52–60.
